# A novel botanical formula prevents diabetes by improving insulin resistance

**DOI:** 10.1186/s12906-017-1848-3

**Published:** 2017-07-05

**Authors:** Juntao Kan, Rodney A. Velliquette, Kerry Grann, Charlie R. Burns, Jeff Scholten, Feng Tian, Qi Zhang, Min Gui

**Affiliations:** 1 Nutrilite Health Institute, 720 Cailun Road, Shanghai, 201203 China; 2 Nutrilite Health Institute, 7575 East Fulton Avenue, Ada, MI 49355 USA

**Keywords:** Botanical test formula, Type 2 diabetes mellitus, Insulin resistance, Alloxan, High fat diet, GLUT4, Fenugreek seed, Mulberry leaf, American ginseng

## Abstract

**Background:**

Type 2 diabetes mellitus (T2DM) is a major risk factor for cardiovascular disease, and the prevalence has increased significantly in recent decades to epidemic proportions in China. Individually, fenugreek (*Trigonella foenum graecum*) seed, mulberry (*Morus alba L.*) leaf and American ginseng (*Panax quinquefolius*) root can improve glycemia in various animal models and humans with impaired glucose metabolism and T2DM. The aim of this study was to design an optimized botanical formula containing these herbal extracts as a nutritional strategy for the prevention of insulin resistance and T2DM.

**Methods:**

Cell-free α-amylase and α-glucosidase enzyme assays were used to determine inhibitory potential of extracts. Glucose uptake was examined in differentiated human adipocytes using radiolabeled 2-deoxyglucose. Male Sprague Dawley rats were divided and glycemia balanced into 5 groups: two controls (naïve and model) and three doses of the botanical test formula containing standardized fenugreek seed, mulberry leaf and American ginseng extracts (42.33, 84.66 and 169.33 mg/kg BW). Insulin resistance and T2DM was induced by feeding animals a high fat diet and with an alloxan injection. Glucose tolerance was examined by measuring serum glucose levels following an oral glucose load.

**Results:**

Fenugreek seed and mulberry leaf dose dependently inhibited α-amylase (IC50 = 73.2 μg/mL) and α-glucosidase (IC50 = 111.8 ng/mL), respectively. All three botanical extracts improved insulin sensitivity and glucose uptake in human adipocytes, which lead to the design of an optimized botanical test formula. In a rat model of insulin resistance and T2DM, the optimized botanical test formula improved fasting serum glucose levels, fasting insulin resistance and the development of impaired glucose tolerance. The reduction in epididymal adipose tissue GLUT4 and PDK1 expression induced by high fat diet and alloxan was blunted by the botanical test formula.

**Conclusions:**

A novel botanical formula containing standardized extracts of mulberry leaf, fenugreek seed and American ginseng at a ratio of 1:1.3:3.4 prevented the development of insulin resistance, impaired glucose tolerance and T2DM. Given the rising need for effective non-drug targeting of insulin resistance and progression to T2DM, complementary and alternative nutritional strategies without intolerable side effects could have meaningful impact on metabolic health and diabetes risks.

## Background

Type 2 diabetes mellitus (T2DM) is a major risk factor for cardiovascular disease, which is the leading cause of death in China [1, 2]. The prevalence of T2DM has increased significantly in recent decades and has reached epidemic proportions in China. A Chinese national survey from June 2007 through May 2008 reported the prevalence of diabetes and prediabetes were 9.7% and 15.5%, respectively [3]. Approximately three years later, another survey reported an increase in the prevalence to 11.6% and 50.1%, respectively [2]. While different sampling methods, screening procedures and diagnostic criteria were used, these reports clearly document a rapid increase in risk and indicate a need for natural, botanical strategies to prevent the development of T2DM in the Chinese population.

Botanicals have been used in traditional Chinese medicine (TCM) for thousands of years to treat T2DM, which is named “wasting-thirst” in TCM. Complementary and alternative therapies uses for prevention and treatment of T2DM are now common practices throughout the world [4–8]. Plants provide a rich source of phytochemicals that have meaningful potential to be used as alternatives to or with western drug treatments [9, 10]. In fact, hundreds of plants either individually or as recipes have been claimed to prevent and or treat T2DM [11]. Of these plants, fenugreek (*Trigonella foenum graecum*) seed, mulberry (*Morus alba L.*) leaf and American ginseng (*Panax quinquefolius*) root are frequently reported as efficacious (see reviews) [12–14].

Fenugreek is one of the oldest medicinal plants, originating in India and Northern Africa. The use of fenugreek for medicinal and culinary uses dates back as far back as six thousand years [15]. Some of the first western scientific publications on fenugreek seed in preventing and treating diabetes appeared over 40 years ago [16–22]. A major phytochemical component of fenugreek seed, 4-hydroxyisoleucine (4-OH-IIe), has also been used in the treatment of diabetes [23–26]. A mechanism of action attributed to the efficacy of 4-OH-IIe is stimulating glucose-dependent insulin secretion from pancreatic beta cells [25–27]. Fenugreek seed has also been combined with sulfonylureas and shown to further improve fasting and post-prandial glycemic control in T2DM patients [28].

Dietary intake of mulberry leaf extract has been shown to improve glucose homeostasis in various diabetic animal models [29–33]. Many of these reports suggest that 1-deoxynojirimycin (DNJ), the most abundant iminosugar in mulberry leaf, to be a major phytochemical component contributing to the efficacy. The most noted mechanism of action of DNJ is as an alpha-glucosidase inhibitor [34]. Mulberry leaf extract and DNJ have also been reported to improve postprandial glycemic response in individuals with impaired glucose metabolism [35–37] and those with T2DM [38].

Ginseng is traditionally used as a Qi-tonifying herb and is often considered to be a panacea (cure-all and longevity). Asian (*Panax ginseng*) and American (*Panax quinquefolius*) ginseng root are the main varieties consumed. Triterpene saponins (ginsenosides) are the major bioactive components of ginseng root [39]. The American variety of ginseng has become increasingly popular as a natural health product, owning to its various pharmacological properties. American ginseng has been shown to improve the diabetes phenotype or insulin resistance in animal models [40–42], as well as in humans with and without T2DM [13, 43–45].

Given fenugreek seed, mulberry leaf and American ginseng have individually been reported to be efficacious in improving glucose homeostasis, our objective was to design a unique botanical formula containing a combination of standardized fenugreek seed, mulberry leaf and American ginseng extracts that had the potential to synergize each other in the prevention of insulin resistance and T2DM. We first examined the in vitro efficacy of fenugreek seed, mulberry leaf and American ginseng extracts which lead to the design and testing of an optimized botanical formula for its preventive properties in an animal model of insulin resistance and T2DM.

## Methods

### α-amylase assay

A 96-well, cell-free α-amylase assay was established by using 2-chloro-p-nitrophenyl-α-D-maltotrioside (CNPG3) as a substrate in an assay buffer which was prepared by adding 14.5 g sodium chloride, 0.15 g calcium chloride dihydrate and 20 g of non-fat dry milk in 1 L deionized water, pH 5.0. Twenty-five μL of each extract were premixed with 25 μL of α-amylase (1 U/mL) and incubated for 30 min at room temperature. Then 200 μL of 37 °C CNPG3 was added to all wells and the plate was immediately read at 405 nm and every 30 s for 5 min at 37 °C using a Spectramax plate reader (Molecular Devices, Sunnyvale, CA). Acarbose served as positive control (100 μM) and defined 100% inhibition. All reagents, chemicals and enzyme were purchased from Sigma-Aldrich Corporation (St. Louis, MO).

### α-Glucosidase assay

A 96-well, cell-free α-glucosidase assay was established using rat intestinal acetone powder as the enzyme source and sucrose as the substrate. The reaction was carried out by adding 60 μL of sucrose (20 mM in water), 30 μL of extract (in water at 4X of final dose tested) and 30 μL of enzyme solution (3.75 mg/mL in a buffer of 15 mM potassium phosphate and 30 mM sodium phosphate, pH 7.0) to each well, sealed and incubated for 25 min at 37 °C. Following incubation, 120 μL of glucose assay reagent (Wako Chemicals, Tokyo, Japan) was added to each well, sealed and incubated for 5 min at 37 °C. Absorbance was then read in a Spectramax plate reader at 510 nM (Molecular Devices, Sunnyvale, CA). 1-deoxynojirimycin hydrochloride served as positive control (50 μM) and defined 100% inhibition. All other reagents, chemicals and enzyme were purchased from Sigma-Aldrich Corporation (St. Louis, MO).

### Glucose uptake assay

The assay was performed by Zen-Bio Inc., (Research Triangle Park, NC), using primary human subcutaneous adipocytes that were differentiated in a 96-well format at 37 °C with 5% CO_2_. Two weeks after cells were differentiated, they were serum starved in the presence of extracts for 20 h. Following the treatment, 1 nM insulin and a cocktail containing 2-deoxyglucose (2-DOG) and ^3^H-2-DOG were applied to cells and allowed to incubate for 2 h. Cells were then washed with phosphate buffered saline and lysed, and glucose uptake was determined as counts/well. Cytochalasin B (10 μM) served as a non-specific 2-DOG uptake to account for non-insulin induced glucose uptake. Change in insulin sensitivity was calculated by setting the effect of 1 nM insulin as 0% and 100 nM insulin as 100%.

### Botanical test formula preparation

The botanical test formula was prepared by Nutrilite Health Institute through mixture of commercially available fenugreek seed extract (from India, extracted with 55% to 75% ethanol and standardized to 4.5% 4-OH-IIe), mulberry leaf extract (from China, extracted with water and standardized to 1.25% DNJ) and American ginseng extract (from USA, extracted with water and standardized to 5.5% ginsenoside). The botanical test formula contained 88 mg of fenugreek seed extract, 120 mg of mulberry leaf extract and 300 mg of American ginseng extract.

### Animal model and treatment

Male Sprague Dawley rats (150–200 g) were purchased from Charles River (Beijing, China). Animals were housed in a temperature and humidity-controlled room (22–23 °C and 46–63%, respectively) and had free access to food and water. Blood samples were taken from the tail vein and all animals received humane care. The experimental protocols were approved by the Institutional Animal Care and Use Committee of Beijing Institute for Drug Control.

A T2DM model was established by following the protocol from the China Food and Drug Administration for health food registration with the claim of “Assist in Reducing Blood Glucose Level” [46]. After one-week acclimation on a standard control chow diet, the animals were fasted 3 h and levels of serum glucose and postprandial serum glucose at 0.5 h after an oral glucose load (2.5 g/kg) were measured to establish baseline glycemia. Animals were glycemia balanced and divided into 5 groups: 1) naïve control (*n* = 10); 2) model control (*n* = 10); 3) 42.33 mg/kg body weight (BW) (1X) (*n* = 10); 4) 84.66 mg/kg BW (2X) (*n* = 9); 5) 169.33 mg/kg BW (4X) (*n* = 7) of the test formula. Model control and treatment groups were given either vehicle or test formula by daily oral gavage for 33 d. After one-week of treatment (day 7), both model control and treatment groups were fed a high fat diet (HFD) (Charles River) for an additional three weeks. HFD was comprised of control chow diet (52.6% *w*/w) plus lard (10% *w*/w), sucrose (15% *w*/w), egg yolk powder (15% *w*/w), casein (5% *w*/w), sodium cholate (0.2% *w*/w), calcium bicarbonate (0.6% *w*/w), calcium carbonate (0.4% *w*/w) and cholesterol (1.2% *w*/w). After three weeks of HFD, animals were fasted for 24 h and a single i.p. injection of alloxan (103–105 mg/kg) was given to induce hyperglycemia. Animals continued the HFD and botanical test formula for another 4 d. Animals were then fasted for 3 h and oral glucose tolerance test (OGTT) was performed. Blood was collected at baseline (0 h) for determination of the serum glucose, insulin, triglyceride and total cholesterol, and at 0.5 h and 2 h after OGTT (2.5 g/kg) for postprandial serum glucose. Serum glucose area under the curve (AUC) was calculated by the following equation, where SG = serum glucose:

$$ \mathrm{AUC}=\frac{\left(0\mathrm{h}\kern0.24em \mathrm{SG}+0.5\mathrm{h}\kern0.24em \mathrm{SG}\right)\times 0.5}{2}+\frac{\left(0.5\mathrm{h}\kern0.24em \mathrm{SG}+2\mathrm{h}\kern0.24em \mathrm{SG}\right)\times 1.5}{2} $$

### Assessment of insulin resistance index using HOMA-IR

Fasting insulin resistance index was assessed using the homeostasis model assessment for insulin resistance (HOMA-IR) originally described by Mathew [47]. HOMA-IR was calculated using the following formula:

HOMA IR = fasting glucose (mmol/L) × fasting insulin (μU/mL)/22.5.

### Serum biomarker analysis

Serum glucose, total cholesterol, triglycerides (BioSino, Beijing, China) and insulin levels (Jiancheng, Nanjing, China) were determined with commercially available kits in a 96-well format per manufacturer’s instructions. In brief, the glucose assay kit uses an enzyme mix that oxidizes glucose to generate a product that reacts with a dye to generate color that is read in a plate reader at 570 nm. The total cholesterol assay kit uses a mix of enzymes to first hydrolyze cholesterol esters followed by oxidation of the free cholesterol to produce hydrogen peroxide that reacts with a probe to produce color that is read in a plate reader at 570 nm. In the triglyceride assay kit, samples are exposed to lipases that generate free fatty acids and glycerol. The glycerol is then oxidized to generate a product which reacts with the probe to generate color that is read in a plate reader at 570 nm.

The insulin kit is a sandwich enzyme linked immunosorbent assay. The principles of the assay are to capture the insulin molecules from samples in the wells of a microtiter plate coated by pre-titered amount of a monoclonal mouse anti-rat insulin antibody and then the binding of biotinylated polyclonal antibodies to the captured insulin. Wells are then washed to remove unbound materials. Next, horseradish peroxidase is added to wells to bind to the immobilized biotinylated antibodies. The wells are then washed to remove any free enzyme conjugates, and quantification of immobilized antibody-enzyme conjugates are determined by horseradish peroxidase activities in the presence of the substrate 3,3′,5,5′-tetramethylbenzidine. The enzyme activity is measured spectrophotometrically by the increased absorbency at 450 nm, corrected from the absorbency at 590 nm, after acidification of formed products.

### Western blot

Epididymal adipose tissue was homogenized in lysis buffer, which was comprised of 50 mM Tris-HCl (pH 7.4), 0.1% sodium dodecyl sulfate (SDS), 2 M phenylmethylsulfonyl fluoride, 10 g/mL leupeptin (Amresco Solon, OH) and boiled in 5× loading buffer (Beyotime Shanghai, China). Twenty μg of protein was then separated by SDS-PAGE and transferred to 0.45 μm polyvinylidene difluoride membrane (Millipore, Billerica, MA). After transfer, membranes were blocked in 5% non-fat powdered milk in phosphate buffered saline with 0.1% Tween 20 and probed with 3-phosphoinositide-dependent kinase-1 (PDK1) (Cell Signaling, Danvers, MA), glucose transporter 4 (GlUT4), (Santa Cruz Biotechnology, Santa Cruz, CA) and glyceraldehyde 3-phosphate dehydrogenase (GAPDH) (Tdybio Beijing, China). Horseradish peroxidase-conjugated secondary antibodies and a chemiluminescence substrate kit were used in detection of specific proteins (Millipore, Billerica, MA).

### Statistical analysis

Quantitative data are presented as mean ± standard deviation (SD). Comparisons between groups were made using two-way repeated measures ANOVA with Holm-Sidak’s multiple comparisons test or one-way ANOVA with Dunnett’s multiple comparison test using GraphPad Prism version 6.00 for Windows (GraphPad Software, La Jolla, CA, www.graphpad.com). A significant effect was defined as *P* < 0.05.

## Results

### Effect on carbohydrate digestive enzymes

**Fig. 1:**
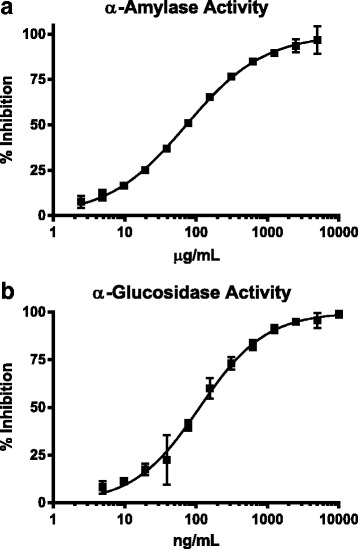
The effect of fenugreek seed (FS) and mulberry leaf (ML) on carbohydrate digestive enzymes. The effect of fenugreek seed on α-amylase (**a**) and mulberry leaf on α-glucosidase (**b**) activity were determined in a cell-free, enzyme-substrate assay model. Data were fitted with log (inhibitor) vs. response, variable slope (four parameters) equation in Prism Graph Pad 6.00. Data are presented as mean ± SD of triplicates

Fenugreek seed and mulberry leaf extracts concentration dependently inhibited α-amylase (IC50 = 73.2 μg/mL) and α-glucosidase (IC50 = 111.8 ng/mL), respectively (Fig. 1a, b). American ginseng extract had no inhibitory activity on either enzyme, fenugreek seed extract did not inhibit α-glucosidase and mulberry leaf extract did not inhibit α-amylase (data not shown).

### Effect on glucose uptake and insulin sensitively

**Fig. 2:**
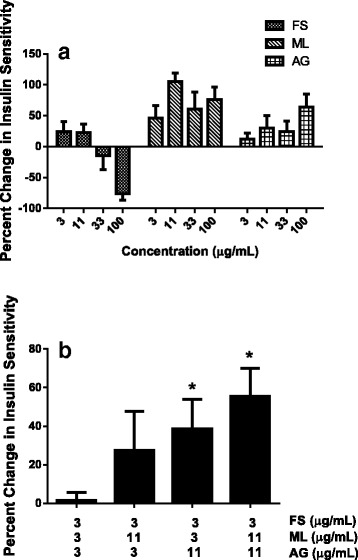
The individual (**a**) and synergetic (**b**) effect of fenugreek seed (FS), mulberry leaf (ML) and American ginseng (AG) extracts on insulin sensitivity in differentiated primary human subcutaneous adipocytes. After 20 h of pre-treatment with extracts or botanical test formula, glucose uptake was initiated with the addition of a cocktail containing 2-DOG and ^3^H-2-DOG with 1 nM insulin and incubate for 2 h. Change in insulin sensitivity was determined by setting 1 nM insulin as 0% and 100 nM as 100%, and calculating the percent change in the delta between 1 nM and 100 nM insulin. Data are presented as mean ± SD of triplicates. ^*^ *P* < 0.05 vs. 1:1:1 ratio group

All extracts at lower doses, 3 and 11 μg/mL, were effective at increasing glucose uptake and insulin sensitivity at submaximal insulin levels (Fig. 2a). Mulberry leaf and American ginseng maintained effectiveness at higher doses (33 and 100 μg/mL). Higher doses of fenugreek seed caused cells to detach from the plate which were lost during the washing step, resulting in a negative effect on glucose uptake and insulin sensitivity (Fig. 2a). Examination of low doses of test formulas at different ratios of fenugreek seed: mulberry leaf: American ginseng resulted in a significant increase in insulin sensitivity at ratios of 1:1:3.6 and 1:3.6:3.6, compared to 1:1:1 (Fig. 2b; *P* < 0.05). Based on these data, commercial cost viability and regulatory limits on doses, an optimized test formula was designed by combining fenugreek seed, mulberry leaf and American ginseng at a ratio of 1:1.3:3.4.

### Effect of test formula on the development of insulin resistance and T2DM in an animal model

**Fig. 3:**
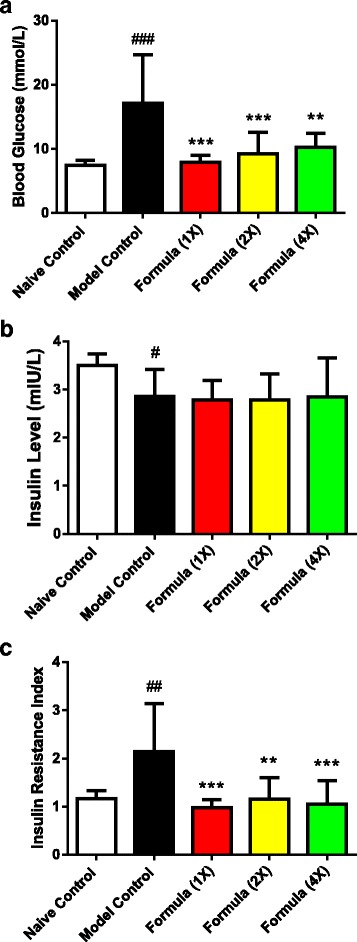
The effect of the botanical test formula on fasting glycemia status. Fasting (3 h) serum glucose (**a**), insulin (**b**) and HOMA-IR (**c**) in a chronic HFD fed and an acute alloxan induced model of insulin resistance and T2DM treated with the botanical test formula comprised of fenugreek seed, mulberry leaf and American ginseng for 33 d. Naïve control (*n* = 10); model control (*n* = 10); 1× (*n* = 10); 2× (*n* = 9); 4× (*n* = 7). Data are presented as mean ± SD. ^###^ *P* < 0.001, ^##^ *P* < 0.01 and ^#^ *P* < 0.05 vs. naïve control group, ^***^ *P* < 0.001 and ^**^ *P* < 0.01 vs. model control group

Fasting serum glucose was significantly higher, 17.1 mmol/L ± 7.5 vs 7.5 mmol/L ± 0.75 (Fig. 3a; *P* < 0.001), while insulin was lower, 2.9 mIU/L ± 0.56 vs 3.5 mIU/L ± 0.24 (Fig. 3b; *P* < 0.05) in model control compared to naïve control. This resulted in a significant elevation in HOMA-IR, which was nearly doubled (2.1 ± 0.99 vs. 1.2 ± 0.16; Fig. 3c). After 33 d of treatment with test formula, the induction of insulin resistance and T2DM with an alloxan injection was prevented at all doses. Both fasting serum glucose and HOMA-IR values were similar to naïve control and significantly lower when compared to model control (Fig. 3a, c). These effects were achieved without a change in fasting insulin compared to model (Fig. 3b). In addition, maximal efficacy appeared to be present at the lowest dose tested, as higher doses resulted in equal efficacy.

**Fig. 4:**
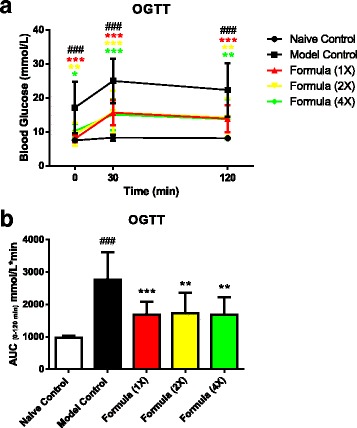
The effect of the botanical test formula on oral glucose tolerance. Serum glucose levels (**a**) and AUC (**b**) in a chronic HFD fed and an acute alloxan induced model of insulin resistance and T2DM treated with the botanical test formula comprised of fenugreek seed, mulberry leaf and American ginseng for 33 d. Animals were fasted 3 h followed by an administration of an oral glucose load (2.5 g/kg) and blood was collected at 0, and 0.5 and 2 h post challenge. Naïve control (*n* = 10); model control (*n* = 10); 1× (*n* = 10); 2× (*n* = 9); 4× (*n* = 7). Data are presented as mean ± SD. ^###^ *P* < 0.001 vs. naïve control group, ^***^ *P* < 0.001, ^**^ *P* < 0.01 and ^*^ *P* < 0.05 vs. model control group

Postprandial serum glucose levels at 0.5 h and 2 h after glucose challenge and AUC were significantly higher in model control compared with naïve control group (Fig. 4; *P* < 0.001). All doses of the test formula significantly improved postprandial serum glucose (Fig. 4a) resulting in a significantly lower AUC, compared to model control (Fig. 4b).

**Fig. 5:**
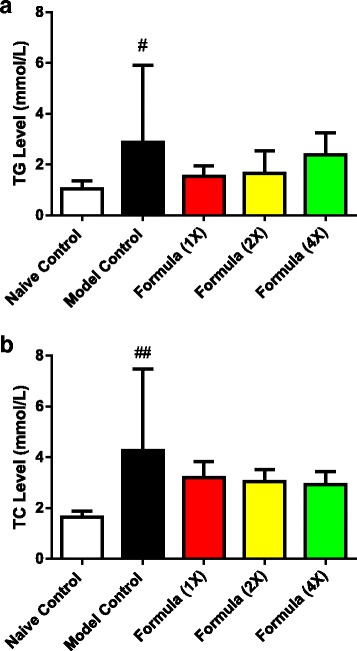
The effect of the botanical test formula on fasting lipids. Fasting (3 h) triglycerides (**a**) and total cholesterol (**b**) levels in a chronic HFD fed and an acute alloxan induced model of insulin resistance and T2DM treated with a test formula comprised of fenugreek seed, mulberry leaf and American ginseng for 33 d. Naïve control (*n* = 10); model control (*n* = 10); 1× (*n* = 10); 2× (*n* = 9); 4× (*n* = 7). Data are presented as mean ± SD. ^###^ *P* = 0.0015 vs. naïve control group, ^#^ *P* < 0.05 vs. naïve control group

Serum triglyceride and total cholesterol levels were significantly elevated in model control compared to naïve control, indicating that lipid metabolism was dysfunctional in this model (Fig. 5). However, while values tended to be lower, none of the botanical test formula doses significantly affected serum triglyceride and total cholesterol levels.

**Fig. 6:**
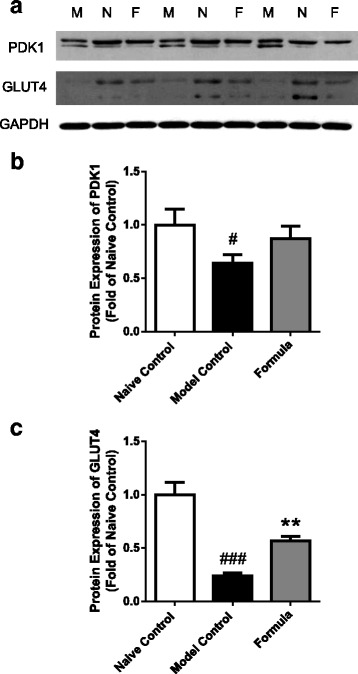
The effect of the test formula (1X dose) on adipose tissue expression of PDK1 and GlUT4. Western blot (**a**) and densitometry analysis of PDK1 (**b**) and GlUT4 (**c**) in epididymal adipose tissue in a chronic HFD fed and an acute alloxan induced model of insulin resistance and T2DM treated with the botanical test formula comprised of fenugreek seed, mulberry leaf and American ginseng for 33 d. GAPDH was used as the loading control. Quantitated data are presented as mean ± SD of pool group samples run in triplicate. Model control (M); Naïve control (N); Test Formula (1×) (F). ^###^ *P* < 0.001 and ^#^ *P* < 0.05 vs. naïve control group, ^**^ *P* < 0.01 vs. model control group

Both PDK1 and GlUT 4 were significantly reduced in model control compared to naïve control (*P* < 0.05 and *P* < 0.001, respectively), and the botanical test formula blunted this reduction (Fig. 6).

## Discussion

Insulin resistance, defined as either impaired fasting glycemia, hyperinsulinemia or both, is a typical metabolic state that occurs prior to the development of T2DM. Targeting insulin resistance and glucose uptake is usually a first line treatment strategy when medically treating diabetes [1]. Liver and adipose tissue are the main targets of T2DM medications like metformin and thiazolidinediones [48]. An additional strategy used to manage postprandial glycemia is inhibiting the hydrolysis of dietary oligosaccharides in the gastrointestinal track with α-amylase and α-glucoside inhibitors [49, 50].

Individually, fenugreek seed, mulberry leaf and American ginseng have been shown to have glucose modulating effects in healthy prediabetic and T2DM subjects [23, 24, 28, 35–37, 43, 44, 51]. Here in, we present both in vitro and in vivo data on a novel formula which contains these botanical extracts in a specific ratio. The botanical test formula significantly improved adipocyte insulin sensitivity, glucose uptake, and in an animal model, prevented the development of insulin resistance and T2DM. Diabetes prevention was observed despite lower fasting serum insulin, suggesting the botanical test formula specifically preserved insulin sensitivity.

Components of all three herbal extracts used here in have been reported to improve glucose uptake in various cell lines. Mulberry leaf has been reported to increase glucose uptake in primary adipocytes [52], DNJ increased glucose uptake in 3 T3-L1 adipocytes [53] and 4-OH-IIe from fenugreek seed increased glucose uptake in L6-GLUT4myc myotubes [54]. Ginsenoside Rb1 from American ginseng was also shown to stimulate basal and insulin-mediated glucose uptake in 3 T3-L1 adipocytes and C2C12 myotubes [55]. We have shown that fenugreek seed, mulberry leaf and American ginseng increased insulin sensitivity in human adipocytes, and resulted in an increase in glucose uptake under low, submaximal insulin levels. We optimized the ratio of fenugreek seed, mulberry leaf and American ginseng in the botanical test formula to 1:1:3.6 which significantly increased insulin sensitivity compared with a test formula consisting of 1:1:1 ratio. More importantly, the botanical test formula consisting of a similar ratio (1:1.3:3.4) prevented the development of T2DM in an animal model by maintaining insulin sensitivity under reduced insulin levels.

Glucose transporters (GLUT) are a family of membrane proteins that transport glucose across the plasma membrane. Among them, GLUT4 is specifically located in adipose tissue and striated muscles and is regulated by insulin and post insulin receptor signaling molecules [56–58]. PDK1 is a critical kinase required for insulin-induced GLUT4 translocation and glucose uptake [59–61]. PDK1 acts as an upstream protein kinase phosphorylating and activating several AGC-family members implicated in the control of cell metabolism [62]. The reduction in adipose tissue expression of PDK1 and GLUT4 in our study was significantly blunted by the botanical test formula. While fenugreek seed, mulberry leaf and American ginseng individually have been shown to increase the expression of GLUT4 in 3 T3-L1 adipocytes and in insulin resistant animal models [52–54, 63, 64], a PDK1/GLUT4-mediated mechanism has not been previously described for a formula containing these herbal extracts.

Carbohydrate digestion and absorption is an important factor involved in determining postprandial blood glucose levels. Digestive enzymes, α-amylase and α-glucosidase play vital roles in carbohydrate digestion of oligosaccharides to di and monosaccharides. It was reported that fenugreek seed was able to inhibit α-amylase activity in dose-dependent manner, while mulberry leaf was a potent α-glucosidase inhibitor [24, 51, 65–67]. Compared with acarbose, a potent α-amylase and α-glucosidase inhibitor drug used to treat T2DM, mulberry leaf extract has been reported to be effective with fewer side effects, such as abdominal flatulence and distention [67]. Our botanical test formula included a combination of fenugreek seed and mulberry leaf to target both α-amylase and α-glucosidase for more effective modulation of dietary carbohydrate digestion.

## Conclusions

We have demonstrated in our research the in vivo prevention of insulin resistance and T2DM with a novel botanical formula consisting of standardized fenugreek seed, mulberry leaf and American ginseng extracts at a specific ratio. We have also identified some potential mechanisms involved which include inhibition of the oligosaccharide digestive enzymes, α-amylase and α-glucosidase, maintenance of molecular insulin sensitivity and improved glucose uptake under reduced or low insulin conditions. Given the rising need for effective non-drug interventions targeting insulin resistance and maintenance of healthy glucose levels, effective commercially available dietary supplements with minimal side effects would have meaningful impact on metabolic health and risks. Our data indicate that a dietary supplement containing fenugreek seed, mulberry leaf and American ginseng could be a commercially viable alternative for this purpose and additional testing in a human clinical trial is warranted.

## Abbreviations

GLUT4: Glucose transporter 4
HFD: High fat diet
HOMA-IR: Homeostasis model assessment for insulin resistance
PDK1: 3-phosphoinositide-dependent kinase-1
T2DM: Type 2 diabetes mellitus
